# The composition of the γ-secretase complex defines its Aβ product profile

**DOI:** 10.1186/1750-1326-8-S1-P2

**Published:** 2013-09-13

**Authors:** Hermien Acx, Lucía Chávez Gutiérrez, Lutgarde Serneels, Bart De Strooper

**Affiliations:** 1 VIB Center for the Biology of Disease, Leuven, Belgium; 2 Center for Human Genetics, KU Leuven, Leuven, Belgium

## Background

Aβ peptides accumulate and aggregate in the brain of patients suffering from Alzheimer’s disease (AD). Since γ-secretase is the final protease involved in the production of Aβ peptides, it has been proposed as a potential drug target in AD. This multiprotein complex consists of four essential subunits: presenilin (PSEN), nicastrin (NCT), anterior pharynx defective (APH1) and presenilin enhancer 2 (PEN-2), which assemble in a 1:1:1:1 stoichiometry. As two PSEN genes and two APH1 genes exist, at least four different γ-secretase complexes exist. Previous studies suggest that this structural heterogeneity has functional implications [1]. Here, we show that the subunit composition of the γ-secretase complex determines its activity and we unravel the biochemical mechanism underlying these differences.

## Materials and methods

The activity of purified γ-secretase complexes was assessed in an *in vitro* assay. The endopeptidase and carboxypeptidase-like activities of the γ-secretase complex were evaluated by measuring the *de novo* generation of amyloid precursor protein intracellular domain (AICD) or the conversion of Aβ43/Aβ42 into Aβ40/Aβ38, respectively [2]. To confirm our results on a cell based level, we measured the Aβ peptides secreted in the medium by mouse embryonic fibroblasts expressing only one type of γ-secretase complex.

## Results

PSEN2 containing complexes lower the overall activity of the γ-secretase, relative to the corresponding PSEN1 complexes. In contrast, APH1B-containing γ-secretase complexes did not change endopeptidase activity levels but reduce the efficiency of the carboxypeptidase-like activity, when compared to the corresponding APH1 A-containing complexes. Interestingly, the effect observed in the APH1B-containing γ-secretase complexes is similar to the reported familial AD PSEN mutations(2) and suggests that APH1B-containing complexes are characterized by a more rapid product release, which explains why more longer and aggregation prone Aβ peptides are generated by APH1B complexes.

## Conclusions

Taken all together our results show that the composition of the γ-secretase complex defines distinctive Aβ product profiles and supports that specific targeting of APH1B-containing γ-secretase complexes may represent a valid strategy in Alzheimer’s disease therapy.
